# Risk Factors for 30-Day Readmission and Reoperation Following Clavicle Open Reduction Internal Fixation

**DOI:** 10.7759/cureus.76632

**Published:** 2024-12-30

**Authors:** Richelle Fassler, Nishanth Muthusamy, Lekha Yaramada, Kenny Ling, David Komatsu, Edward D Wang

**Affiliations:** 1 Orthopaedic Surgery, Stony Brook University Hospital, Stony Brook, USA; 2 Medicine, Renaissance School of Medicine, Stony Brook University Hospital, Stony Brook, USA

**Keywords:** 30-day readmission, american college of surgeons national surgical quality improvement program, clavicle fractures, open reduction internal fixation, reoperation rate

## Abstract

Introduction

Clavicle open reduction internal fixation (ORIF) is an effective treatment for the surgical management of clavicle fractures. However, the literature surrounding the risk factors for readmission and reoperation following clavicle ORIF remains understudied. The purpose of this study is to investigate the specific risk factors for 30-day readmission and reoperation following clavicle ORIF.

Methods

The American College of Surgeons National Surgical Quality Improvement Program (ACS-NSQIP) database was queried for all patients between 2015 and 2020 who underwent clavicle ORIF. Patients were divided into cohorts, both for readmission and reoperation status, after exclusion criteria. Bivariate logistic regression was used to identify patient demographics and comorbidities associated with readmission and reoperation. Multivariate logistic regression, adjusted for all significant patient demographics and comorbidities, was used to identify the risk factors independently associated with 30-day readmission and reoperation following clavicle ORIF.

Results

A total of 6,132 patients remained after exclusion criteria, with a readmission rate of 0.85% and a reoperation rate of 1.2%. On multivariate analysis, age 40-64 (odds ratio (OR) 2.79, 95% confidence interval (CI) 1.38-5.63; p = 0.004), age 65-74 (OR 3.07, 95% CI 1.00-9.41; p = 0.049), age ≥75 (OR 4.90, 95% CI 1.13-21.22; p = 0.033), American Society of Anesthesiologists (ASA) ≥3 (OR 2.71, 95% CI 1.26-5.37; p = 0.004), and smoking (OR 3.27, 95% CI 1.83-5.87; p < 0.001) were found to be independent risk factors for 30-day readmission. Additionally, age 40-64 years (OR 2.65, 95% CI 1.48-4.78; p = 0.001), age 65-74 (OR 3.51, 95% CI 1.44-8.57; p = 0.006), ASA ≥3 (OR 3.26, 95% CI 1.85-5.77; p < 0.001), and smoking (OR 2.84, 95% CI 1.74-4.65; p < 0.001) were found to be independent risk factors for 30-day reoperation.

Conclusion

Age ≥40 years, ASA ≥3, and smoking were identified as independent risk factors for 30-day readmission. Age 40-74 years, ASA ≥3, and smoking were identified as independent risk factors for 30-day reoperation. These results can guide physicians in preoperative patient counseling and management.

Level of evidence

Level III, retrospective cohort study

## Introduction

Clavicle fractures commonly result from trauma, such as a fall onto the shoulder or outstretched hand, motor vehicle accidents, and sports-related injuries [1]. These fractures are more common in children than in adults, and they can represent more than half of all shoulder fractures [2,3]. Non-displaced clavicle fractures are often treated non-operatively in a sling, but recent studies have suggested higher rates of impaired shoulder strength and decreased range of motion [4,5]. Displaced fractures with minimal cortical contact can be better aligned and stabilized with surgical intervention utilizing open reduction internal fixation (ORIF) [6-8]. Furthermore, the rate of surgical intervention following clavicle fracture has been on the rise, from 6.4% in 2014 to 31.5% in 2018 [4-6].

Despite this increase in surgical intervention, some studies have reported complication rates as high as 37%. Some complications associated with clavicle ORIF include brachial plexus symptoms, hardware irritation, infection, dehiscence, sensory deficits, pain and stiffness, and delayed union [4,9]. Higher rates of complications have also been reported in female patients, along with those with increased age, diabetes, dependent functional status, daily alcohol use, and pulmonary, cardiac, or neurological comorbidities [10]. Additionally, Leroux et al. found that nearly a quarter of clavicle ORIF patients required reoperation, with indications including implant removal, non-union, deep infection, and malunion [11].

Given the high volume of clavicle fractures and increasing frequency of clavicle ORIF, it is imperative to identify patient comorbidities and demographics associated with adverse outcomes. The purpose of this study was to identify patient demographics and comorbidities associated with 30-day readmission and 30-day reoperation after clavicle ORIF. The study encompasses data collected from 6,132 patient records who underwent clavicle ORIF, sourced from the American College of Surgeons National Surgical Quality Improvement Program (ACS-NSQIP)database [12]. We hypothesized that higher rates of comorbidities are associated with an increased risk of 30-day readmission and reoperation following clavicle ORIF.

## Materials and methods

The ACS-NSQIP database was queried for all patients who underwent clavicle ORIF between 2015 and 2020 [12]. The NSQIP data is acquired and collected by trained Surgical Clinical Reviewers from over 600 hospitals in the United States. Furthermore, it is periodically audited to maintain its accuracy and validity. The ACS-NSQIP database is fully deidentified, therefore making this study exempt from our University’s Institutional Review Board approval.

We used Current Procedural Terminology (CPT) code 23515 to identify patients who underwent clavicle ORIF between 2015 and 2020. Patients younger than age 18 were automatically excluded from the database, and patient cases were additionally excluded for missing information in any of the following variables: weight, height, functional status, discharge destination, and American Society of Anesthesiologists (ASA) classification.

Patient demographics included in the study were age, gender, BMI, ASA classification, functional status, smoking status, and chronic steroid use. Patient comorbidities collected include diabetes, chronic obstructive pulmonary disease (COPD), ascites, congestive heart failure (CHF), hypertension, dialysis, disseminated cancer, open wound/wound infection, unintentional weight loss, and bleeding disorders. Additionally, surgical characteristics such as preoperative transfusion and total operative time were collected.

The 30-day postoperative complications investigated in this study included readmission and reoperation. Reasons for readmission and reoperation were also identified (Tables 1, 2).

**Table 1:** Reasons for 30-day readmission following clavicle ORIF ORIF: Open reduction internal fixation

Reason for Readmission	Number	Percentage
Total	26	100.0%
Surgical site related	2	7.7%
Extremity cellulitis	1	3.8%
Acute postoperative pain	1	3.8%
Non-surgical site related	18	69.2%
Fixation failure	5	19.2%
Neurologic complication	4	15.4%
Pulmonary complication	3	11.5%
Fracture	3	11.5%
Gastrointestinal complication	1	3.8%
Genitourinary complication	1	3.8%
Recurrent dislocation	1	3.8%
Other/Unspecified	6	23.1%

**Table 2:** Reasons for 30-day reoperation following clavicle ORIF ORIF: Open reduction internal fixation

Reason for Reoperation	Number	Percentage
Total	55	100.0%
Related to primary procedure	16	29.1%
Infection	10	18.2%
Wound disruption	3	5.5%
Non-union	2	3.6%
Malunion	1	1.8%
Unrelated to primary procedure	29	52.7%
Fracture	17	30.9%
Fixation failure	9	16.4%
Recurrent dislocation	1	1.8%
Shoulder sprain	1	1.8%
Pulmonary	1	1.8%
Other/Unspecified	10	18.2%

**Figure 1:**
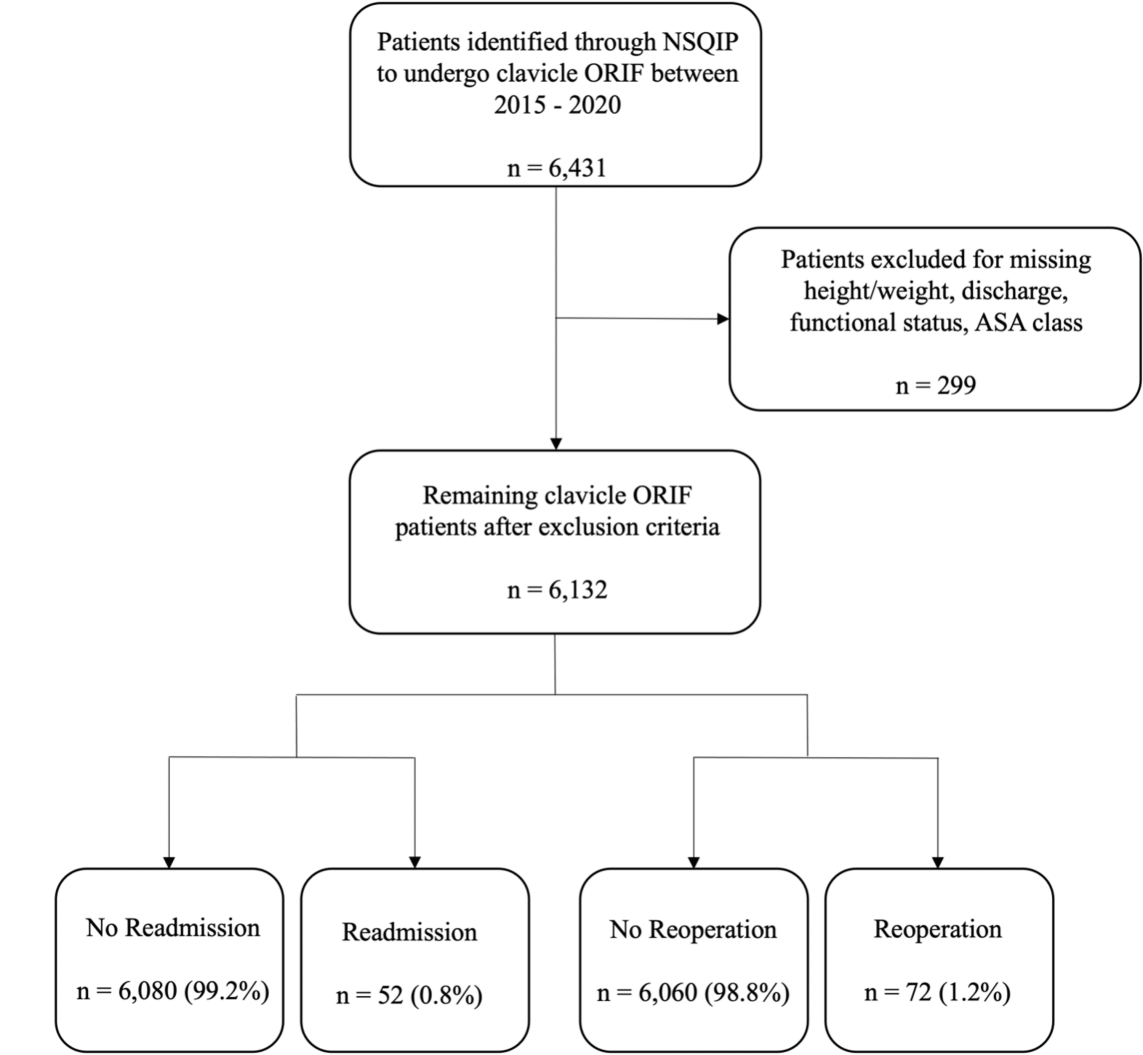
Inclusion and exclusion criteria for readmission and reoperation cohorts among patients undergoing clavicle ORIF NSQIP: National Surgical Quality Improvement Program; ASA: American Society of Anesthesiologists; ORIF: Open reduction internal fixation

A total of 6,431 patients who were treated with clavicle ORIF between 2015 and 2020 were included in this study. Cases were excluded as follows: 228 for missing height or weight, 6 for unknown discharge destination, 56 for unknown functional status, and 9 for missing ASA classification. The remaining 6,132 patients were first divided into cohorts based on 30-day readmission status following clavicle ORIF, with 6,080 (99.2%) in the no readmission cohort and 52 (0.8%) in the readmission cohort. These cases were also divided into cohorts based on 30-day reoperation status following clavicle ORIF, with 6,060 (98.8%) in the no reoperation cohort and 72 (1.2%) in the reoperation cohort (Figure 1). 

All statistical analyses were performed using SPSS Software version 29.0 (IBM Corp., Armonk, USA). Bivariate logistic regression analysis was used to compare patient demographics and comorbidities between cohorts. Multivariate binomial logistic regression, adjusted for all significant demographics and comorbidities, was then used to identify the preoperative risk factors independently associated with readmission and reoperation following clavicle ORIF. Odds ratios (ORs) were reported for 95% confidence intervals (CI). A significant value was set to p < 0.05.

## Results

In this study, we used bivariate analysis to identify patient demographics and comorbidities significantly associated with 30-day readmission following clavicle ORIF, shown in Table 3. The characteristics significantly associated with 30-day readmission were female gender (p = 0.008), age ≥40 years (p < 0.001), ASA ≥3 (p < 0.001), dependent functional status (p = 0.002), smoking (p < 0.001), hypertension (p < 0.001), dialysis (p = 0.002), disseminated cancer (p = 0.004), and 10% weight loss (p = 0.004).

**Table 3:** Patient demographics and comorbidities in patients with and without 30-day readmission following clavicle ORIF p-values were calculated using chi-squared test. Statistical significance was defined as p < 0.05. ASA: American Society of Anesthesiologists; COPD: Chronic obstructive pulmonary disease; CHF: Congestive heart failure; ORIF: Open reduction internal fixation

Characteristic	No Readmission		Readmission		p-value
	Number	Percentage	Number	Percentage	
Total	6,080	100.0%	52	100.0%	
Gender					0.008
Male	4,708	77.4%	32	61.5%	
Female	1,372	22.6%	20	38.5%	
Age					
18-39	3,568	58.7%	13	25.0%	–
40-64	2,124	34.9%	29	55.8%	<0.001
65-74	307	5.0%	6	11.5%	<0.001
≥75	81	1.3%	4	7.7%	<0.001
BMI					
>18.5	106	1.7%	2	3.8%	0.238
18.5-29.9	4902	80.6%	39	75.0%	–
30-34.9	780	12.8%	9	17.3%	0.317
35-39.9	204	3.4%	2	3.8%	0.774
≥40	88	1.4%	0	0.0%	0.997
ASA class					<0.001
1-2	5,440	89.5%	30	57.7%	
≥3	640	10.5%	22	42.3%	
Functional status					0.002
Independent	6,056	99.6%	50	96.2%	
Dependent	24	0.4%	2	3.8%	
Smoking					<0.001
No	4,596	75.6%	26	50.0%	
Yes	1,484	24.4%	26	50.0%	
Diabetes					
No	5,859	96.4%	49	94.2%	–
Non-insulin	138	2.3%	1	1.9%	0.888
Insulin	83	1.4%	2	3.8%	0.147
COPD					0.998
No	6,027	99.1%	52	100.0%	
Yes	53	0.9%	0	0.0%	
Ascites					–
No	6,080	100.0%	52	100.0%	
Yes	0	0.0%	0	0.0%	
CHF					–
No	6,080	100.0%	52	100.0%	
Yes	0	0.0%	0	0.0%	
Hypertension					<0.001
No	5,449	89.6%	37	71.2%	
Yes	631	10.4%	15	28.8%	
Dialysis					0.002
No	6,077	100.0%	51	98.1%	
Yes	3	0.0%	1	1.9%	
Disseminated cancer					0.004
No	6,075	99.9%	51	98.1%	
Yes	5	0.1%	1	1.9%	
Open wound/Wound infection					0.355
No	6,034	99.2%	51	98.1%	
Yes	46	0.8%	1	1.9%	
10% weight loss					0.004
No	6,075	99.9%	51	98.1%	
Yes	5	0.1%	1	1.9%	
Bleeding disorders					0.150
No	6,053	99.6%	51	98.1%	
Yes	27	0.4%	1	1.9%	
Steroid use					0.288
No	6,040	99.3%	51	98.1%	
Yes	40	0.7%	1	1.9%	
Preop transfusion					1.000
No	6,079	100.0%	52	100.0%	
Yes	1	0.0%	0	0.0%	
Operative time					
0-79	2,853	46.9%	27	51.9%	0.472
80-128	2,422	39.8%	17	32.7%	–
≥129	805	13.2%	8	15.4%	0.650

We also used bivariate analysis to identify patient demographics and comorbidities significantly associated with 30-day reoperation following clavicle ORIF, shown in Table 4. The characteristics significantly associated with 30-day reoperation were with female gender (p = 0.003), age ≥ 40 years (p < 0.001), ASA ≥ 3 (p < 0.001), smoking (p < 0.001), COPD (p = 0.006), hypertension (p < 0.001), and disseminated cancer (p = 0.010).

**Table 4:** Patient demographics and comorbidities in patients with and without 30-day reoperation following clavicle ORIF p-values were calculated using chi-squared test. Statistical significance was defined as p < 0.05. ASA: American Society of Anesthesiologists; COPD: Chronic obstructive pulmonary disease; CHF: Congestive heart failure; ORIF: Open reduction internal fixation

Characteristic	No Reoperation		Reoperation		p-value
	Number	Percentage	Number	Percentage	
Total	6,060	100.0%	72	100.0%	
Gender					0.003
Male	4,695	77.5%	45	62.5%	
Female	1,365	22.5%	27	37.5%	
Age					
18-39	3,562	58.8%	19	26.4%	–
40-64	2,112	34.9%	41	56.9%	<0.001
65-74	303	5.0%	10	13.9%	<0.001
≥75	83	1.4%	2	2.8%	0.045
BMI					
>18.5	106	1.7%	2	2.8%	0.431
18.5-29.9	4,889	80.7%	52	72.2%	–
30-34.9	775	12.8%	14	19.4%	0.081
35-39.9	204	3.4%	2	2.8%	0.910
≥40	86	1.4%	2	2.8%	0.283
ASA class					<0.001
1-2	5,428	89.6%	42	58.3%	
≥3	632	10.4%	30	41.7%	
Functional status					0.998
Independent	6,034	99.6%	72	100.0%	
Dependent	26	0.4%	0	0.0%	
Smoking					<0.001
No	4,585	75.7%	37	51.4%	
Yes	1,475	24.3%	35	48.6%	
Diabetes					
No	5,840	96.4%	68	94.4%	–
Non-insulin	138	2.3%	1	1.4%	0.639
Insulin	82	1.4%	3	4.2%	0.970
COPD					
No	6,010	99.2%	69	95.8%	0.006
Yes	50	0.8%	3	4.2%	
Ascites					
No	6,060	100.0%	72	100.0%	
Yes		0.0%		0.0%	
CHF					
No	6,060	100.0%	72	100.0%	
Yes		0.0%		0.0%	
Hypertension					<0.001
No	5,433	89.7%	53	73.6%	
Yes	627	10.3%	19	26.4%	
Dialysis					0.999
No	6,056	99.9%	72	100.0%	
Yes	4	0.1%	0	0.0%	
Disseminated cancer					0.010
No	6,055	99.9%	71	98.6%	
Yes	5	0.1%	1	1.4%	
Open wound/Wound infection					0.067
No	6,015	99.3%	70	97.2%	
Yes	45	0.7%	2	2.8%	
10% weight loss					0.999
No	6,054	99.9%	72	100.0%	
Yes	6	0.1%	0	0.0%	
Bleeding disorders					0.263
No	6,033	99.6%	71	98.6%	
Yes	27	0.4%	1	1.4%	
Steroid use					0.461
No	6,020	99.3%	71	98.6%	
Yes	40	0.7%	1	1.4%	
Preop transfusion					1.000
No	6,059	100.0%	72	100.0%	
Yes	1	0.0%	0	0.0%	
Operative time					
0-79	2,839	46.8%	41	56.9%	0.082
80-128	2,417	39.9%	22	30.6%	–
≥129	804	13.3%	9	12.5%	0.603

We then used multivariate logistic regression to identify which postoperative demographics and comorbidities were independently associated with 30-day readmission and reoperations following clavicle ORIF. We identified age 40-64 (OR 2.79, 95% CI 1.38-5.63; p = 0.004), age 65-74 (OR 3.07, 95% CI 1.00-9.41; p = 0.049), age ≥75 (OR 4.90, 95% CI 1.13-21.22; p = 0.033), ASA ≥3 (OR 2.71, 95% CI 1.26-5.37; p = 0.004), and smoking (OR 3.27, 95% CI 1.83-5.87; p < 0.001) to be independent risk factors for 30-day readmission, shown in Table 5. We also identified age 40-64 years (OR 2.65, 95% CI 1.48-4.78; p = 0.001), age 65-74 (OR 3.51, 95% CI 1.44-8.57; p = 0.006), ASA ≥3 (OR 3.26, 95% CI 1.85-5.77; p < 0.001), and smoking (OR 2.84, 95% CI 1.74-4.65; p < 0.001) to be independent risk factors for 30-day reoperation, shown in Table 6.

**Table 5:** Multivariate analysis of significant patient demographics and comorbidities for 30-day readmission following clavicle ORIF P-values were calculated using logistic regression. Statistical significance was defined as p < 0.05. ASA: American Society of Anesthesiologists; OR: Odds ratio; CI: Confidence interval; ORIF: Open reduction internal fixation

Comorbidities	OR	95% CI	p-value
Gender	1.55	0.85-2.81	0.152
Age			
18-39			–
40-64	2.79	1.38-5.63	0.004
65-74	3.07	1.00-9.41	0.049
≥75	4.9	1.13-21.22	0.033
ASA class	2.71	1.26-5.37	0.004
Functional status	2.3	0.44-12.14	0.326
Current smoker	3.27	1.83-5.87	<0.001
Hypertension	1.08	0.53-2.22	0.835
Dialysis	11.01	0.99-122.68	0.051
Disseminated cancer	7.89	0.83-75.42	0.073
10% weight loss	8.07	0.61-106.59	0.113

**Table 6:** Multivariate analysis of significant patient demographics and comorbidities for 30-day reoperation following clavicle ORIF P-values were calculated using logistic regression. Statistical significance was defined as p < 0.05. ASA: American Society of Anesthesiologists; OR: Odds ratio; CI: Confidence interval; COPD: Chronic obstructive pulmonary disease; ORIF: Open reduction internal fixation

Comorbidities	OR	95% CI	p-value
Gender	1.61	0.975-2.656	0.063
Age			
18-39			–
40-64	2.65	1.48-4.78	0.001
65-74	3.51	1.44-8.57	0.006
≥75	2.07	0.42-10.12	0.369
ASA class	3.26	1.85-5.77	<0.001
Current Smoker	2.84	1.74-4.65	<0.001
COPD	0.93	0.27-3.26	0.914
Hypertension	1.05	0.57-1.93	0.878
Disseminated cancer	5.41	0.58-50.17	0.137

## Discussion

In this retrospective study, we identified risk factors for 30-day readmission and reoperation following clavicle ORIF. We found 30-day readmission to be associated with female gender, age ≥40 years, ASA ≥3, dependent functional status, smoking, hypertension, dialysis, disseminated cancer, and 10% weight loss. We found 30-day reoperation to be associated with female gender, age ≥40 years, ASA ≥3, smoking, COPD, hypertension, and disseminated cancer. After controlling for significant patient demographics and comorbidities, we identified age ≥40 years, ASA ≥3, and smoking to be independent predictors of 30-day readmission and age 40-74 years, ASA ≥3, and smoking to be independent predictors of 30-day reoperation.

Clavicle fractures are common traumatic shoulder girdle injuries that are often treated non-operatively; however, recent studies on non-operative management have demonstrated significantly higher rates of non-union, higher pain scores, and increased patient dissatisfaction compared to surgical intervention [3,5,13-16]. In certain cases of polytrauma, significant shortening, complete displacement, and comminution, clavicle ORIF may be more strongly considered. Recent studies have shown the potential for improved outcomes with this operative management [3,4,17]. Clavicle ORIF may result in quicker union time, fewer non-unions and malunions, increased patient functional outcomes, and greater patient satisfaction with regards to cosmetic appearance of the shoulder [3,4]. As the threshold for surgical intervention of clavicle fractures continues to be investigated, it is imperative to explore the risk factors that influence the incidence of complications following ORIF treatment.

30-day readmission

Under the Affordable Care Act, the Hospital Readmission Reduction Program (HRRP) was established in 2010 to mitigate unnecessary readmissions and reduce overall healthcare costs [18]. The program placed financial penalties on non-compliant hospitals with higher-than-expected readmission rates. While the focus of the program was to reduce readmission rates in the treatment of myocardial infarction, heart failure, and pneumonia, it also showed a statistically significant decrease in surgical readmissions, primarily for total hip and knee arthroplasties [18].

Postoperative readmission has been well-studied in multiple areas of orthopedics. A recent systematic review found that over 75% of orthopedic studies identified multiple comorbidities as significant risk factors for readmission, including increased age, ASA ≥3, elevated BMI, length of stay, and discharge disposition [19]. When focusing on upper extremity surgery, readmission rates have been shown to vary in the literature. For example, following carpometacarpal arthroplasty, Ling et al. found a readmission rate of 0.5%, while another study on the treatment of proximal humerus fractures found a much higher readmission rate of 4.2% [20,21].

Readmission rates following clavicle ORIF are scarcely reported in the literature. A study by Carrillo et al. utilized a large database from 2005 to 2012 and found a 90-day readmission rate of 3.29% [22]. This study reported the most common reason of readmission to be postoperative infection; however, it did not include analysis of common patient comorbidities. A study recently published by Ling et al. queried the NSQIP database for patients who underwent clavicle ORIF between 2015 and 2020 and sought to identify whether smoking is an independent predictor for 30-day readmission [23]. They divided their pool of patients into two cohorts based on smoking status (smoker and non-smokers) and found current smoking status to be significantly associated with readmission rates. However, unlike our study, they did not include an extensive analysis of other potential patient demographics that can independently predict readmission rate, nor did they include indications for readmission. 

This study of 6,132 patients found a 30-day readmission rate of 0.8%, with age ≥40, ASA ≥3, and smoking to be independent risk factors for readmission. These results mirror other orthopedic studies on readmission which also cite increased age and ASA class to be risk factors [19-21]. Furthermore, smoking is a well-established risk factor for postoperative infections, which has been shown to contribute to readmission risk [22,24]. One review found that surgical site infection and wound complications accounted for nearly half of 30-day readmissions following orthopedic surgery cases [19]. However, our study showed that surgical site related indications for readmission accounted for only 7.7% (2/26) of 30-day readmission, while the majority of indications were non-surgical site related such as fixation failure (19.2%; 5/26) and neurological complications (15.4%; 4/26) (Table 1).

30-day reoperation

In this study, we found a reoperation rate of 1.2% within 30 days following clavicle ORIF. A closer look at the significant demographics and comorbidities associated with reoperation can potentially reduce adverse outcomes and unnecessary hospital costs [25].

Multiple studies have reported higher rates of reoperation following clavicle ORIF. Leroux et al. identified 1,350 patients who underwent clavicle ORIF from 2002 to 2010 and found the two-year reoperation rate to be 24.6% [11]. Similarly, Carrillo et al. reported on 334 cases of clavicle ORIF from 2005 to 2012 and found the two-year reoperation rate to be at 15.9% [22]. Furthermore, Ashman et al. also showed a high reoperation rate of 20% in 143 clavicle ORIF patients [26]. The low reoperation rate in our study may be attributed to the limited period of analysis, restricted to the first 30 days, while the aforementioned studies extended their observation window to two years.

When looking at various patient demographics and comorbidities, this study identified age 40-74 years, ASA ≥3, and smoking to be independent predictors of reoperation. Following shoulder surgery, significantly higher complication rates have been documented in patients with ASA ≥3, among other risk factors [10]. Ling et al. used the NSQIP database to report findings of current cigarette use as an independent patient-related risk factor for 30-day reoperations and postoperative deep incisional surgical site infections following clavicle ORIF; however, they did not analyze other common patient demographics or comorbidities that can independently contribute to reoperation [23]. In line with our findings, extensive literature has shown that smoking and increased age are significantly associated with higher incidence of reoperations [27-29]. A study specifically on clavicle ORIF found that smoking significantly increases complication risks such as symptomatic hardware, infection, and wound dehiscence, some of which lead to reoperation [9]. Additionally, some studies cited the main indication of reoperation to be implant removal due to hardware irritation, with a median time to implant removal of 12 months [11]. In our cohort of patients, fractures (30.9%; 17/55) and infection (18.2%; 10/55) were among the main indications for 30-day reoperation (Table 2).

Limitations

This study was limited by the information available through the ACS-NSQIP database. Therefore, we were not able to account for variables such as surgeon experience, fracture pattern, mechanism of injury, disease severity, postoperative rehabilitation, or location of surgery. Furthermore, our data was restricted to the 30-day window allotted by the NSQIP database, and postoperative outcomes after these 30 days were not able to be investigated. Despite these limitations, we used a large national database to investigate which patient demographics and comorbidities are independent risk factors for readmission and reoperation following clavicle ORIF.

## Conclusions

This retrospective study identified age ≥40 years, ASA ≥3, and smoking as independent risk factors for 30-day readmission and 30-day reoperation following ORIF treatment for midshaft clavicle fractures, with ages 40-74 years specifically associated with reoperation risk. As operative fixation of clavicle fractures becomes an increasingly common practice, it is necessary to investigate which preoperative risk factors most contribute to readmission and reoperation. These results can help guide physicians to appropriately counsel patients regarding their preoperative risk factors and ultimately help to minimize hospital costs associated with readmissions and reoperations following clavicle ORIF.
